# Labor Pains Treated with the Simillimum

**Published:** 1895-05

**Authors:** W. E. Everly

**Affiliations:** Glyndon, Minn.


### LABOR PAINS TREATED WITH THE SIMILLIMUM.

Dr. W. E. Everly, Glyndon, Minn.

Case I.—December 26th, was called to attend Mrs. L., a tall, slim Swedish woman, in confinement, with her fourth child.

Found she had been having pains all day, but had ceased about two hours before. She said when she had the pains they began in the back, and passed down the inner side of the thighs.

She being of an irritable nature and very sensitive to pain, led me to think of Cham., which I gave in Φ one drop in a teaspoonful of water; one dose.

Then finding out the condition found the os fully dilated, though rigid. Now I sat down to wait. In about fifteen minutes she had a slight pain; in ten minutes more another, more severe, and in five more another, when the child was born.

In about fifteen minutes the placenta was expelled without trouble; this she said had always been fast before, and had "to be taken," as she expressed it.

She made a good recovery.

Case II.—June 16th, 1894, I was called to attend Mrs. H. in her first confinement. Finding I had arrived too soon, I sat down to await developments. After waiting two or three hours I jokingly said to her that if she did not hurry up she would keep me up all night. She said she had been thinking the some thing. But on getting up to cross the floor I noticed she seemed dizzy.

Previous to this I had noticed her being drowsy; then I began to study my patient. She is a tall, slim, mild, timid patient, light hair. Dullness, drowsiness, dryness, with tardy dilitation, rigid os, led me to think of Gels., which I gave in the 200th, a dose every twenty minutes, until three doses were taken. In about two hours the child was born without the use of instruments or very severe pain. Made a good recovery.

Case III.—Mrs. A., Scandinavian, slim, medium height, red hair, mother of two children, had been in labor thirty-six hours attended by a midwife.

I found her sad, tearful, fears she won't get well. Consolation aggravates, is very much exhausted, pains ceased, had a chill about ten A. M., has felt no movements or pain since. Midwife said the child was dead, and could not be delivered without instruments. I gave her a dose of Nat-mur.$^{200}$ in water, in about fifteen or twenty minutes she began to have pains; three doses twenty minutes apart brought the child alive and all right, with very little assistance.

